# Interprofessional education at four joint medical and welfare universities: A comparison of face-to-face and distance learning

**DOI:** 10.20407/fmj.2025-030

**Published:** 2026-02-28

**Authors:** Sayuri Nakamura, Atsuhiko Ota, Eizou Umezawa, Mihoko Itoh, Miho Miyamoto, Yuki Higashimoto, Fumiaki Horiba, Hiroyuki Kamei, Shigetaka Tomoda, Masatsugu Ohtsuki

**Affiliations:** 1 Faculty of Nursing, School of Health Sciences, Fujita Health University, Toyoake, Aichi, Japan; 2 Department of Public Health, School of Medicine, Fujita Health University, Toyoake, Aichi, Japan; 3 Department of Medical Sciences Education, School of Medical Sciences, Fujita Health University, Toyoake, Aichi, Japan; 4 Faculty of Rehabilitation, School of Health Sciences, Fujita Health University, Toyoake, Aichi, Japan; 5 Department of Clinical Microbiology, School of Medical Sciences, Fujita Health University, Toyoake, Aichi, Japan; 6 Department of Fundamental Education, School of Medical Sciences, Fujita Health University, Toyoake, Aichi, Japan; 7 Faculty of Pharmacy, Meijo University, Nagoya, Aichi, Japan; 8 Department of Operative Dentistry, School of Dentistry, Aichi-Gakuin University, Nagoya, Aichi, Japan; 9 Department of Clinical General Medicine, School of Medicine, Fujita Health University, Toyoake, Aichi, Japan

**Keywords:** Interprofessional education, Face-to-face learning, Distance learning, Educational effectiveness, Team-based learning

## Abstract

**Objective:**

The objective of this study was to compare the effectiveness of face-to-face and distance learning in interprofessional education.

**Methods:**

We conducted a survey using the Japanese version of the Readiness for Interprofessional Learning Scale (RIPLS) to compare face-to-face and distance learning among students from four joint medical and welfare universities participating in interprofessional education (IPE). Faculty members who participated in both face-to-face and distance learning were also surveyed for free descriptions of the advantages and disadvantages of face-to-face and distance learning; their responses were then coded and categorized.

**Results:**

In both face-to-face and distance learning, the RIPLS overall and “teamwork and collaboration” scores significantly increased after class compared to before class. There was a significant increase in “IPE opportunities” in face-to-face learning. There was little change in “uniqueness of profession” before and after class in both face-to-face and distance learning. The difference in the overall scores before and after class was significantly larger in face-to-face learning than in distance learning. The advantages of face-to-face learning included smooth communication and discussions. Distance learning was useful for connecting universities across large distances; however, there were disadvantages, such as less smooth communication and discussions and difficulty maintaining concentration.

**Conclusion:**

Face-to-face learning may be more effective than distance learning. Distance learning had issues with “IPE opportunities,” while both face-to-face and distance classes had issues with “uniqueness of profession.”

## Introduction

Medical and welfare fields face complex and diverse challenges that require collaboration beyond individual specialties. This necessitates effective inter-professional education before graduation.^1,2^ Fujita Health University is a comprehensive medical university with three departments and five faculties. Since its founding in 1971, it has implemented interprofessional assembly education across various departments and faculties. This interprofessional education (IPE) consists of Assembly I in the first year, Assembly II in the second year, Assembly III in the third year, and Assembly IV in the fourth year (Assemblies I–III are required, while Assembly IV is an elective). Assembly I focuses on communication; Assembly II on teamwork; Assembly III on patient-, user-, family-, and community-centered approaches and understanding of professions; and Assembly IV on collaborative practices.

This study focused on Assembly III. Previous research has shown that interprofessional education scores significantly increase after Assembly III compared with those before the class.^3,4^ Further, there is a significant correlation between group-work participation and peer evaluation.^5^ Until 2019, all Assembly III classes were conducted face-to-face. Subsequently, owing to the coronavirus disease (COVID-19) pandemic, traditional face-to-face classes were restricted, and interprofessional education was conducted remotely from 2020 to 2023. Previous research has reported no differences in the evaluations of face-to-face and remote interprofessional education conducted by these four departments.^6^ A study on face-to-face and remote interprofessional education for nursing students reported higher evaluation scores in distance learning than in face-to-face learning classes.^7^ We believe that it is necessary to compare face-to-face and distance learning and verify the educational effectiveness of the interprofessional education that we conducted, involving seven departments from four medical and welfare universities, to improve interprofessional education.

Therefore, in this study we compared the effectiveness of face-to-face and distance learning interprofessional education.

## Methods

### Participants

The study targeted students from seven faculties and four universities in the fields of medicine and welfare, who participated in interprofessional education programs between 2019 and 2023. Those who completed the pre- and post-class questionnaire surveys were included in the analysis. Due to the impact of COVID-19, the 2020 survey was limited to students from Fujita Health University and was therefore excluded from the analysis. We surveyed seven departments: Medicine, Medical Sciences, Health Sciences, Pharmacy, Social Welfare, Dentistry, and Psychological and Physical Sciences.

Additionally, the study targeted teachers who had experience participating in both face-to-face and remote interprofessional education programs.

### Interprofessional education

The theme for all classes from 2019 to 2023 was “Quality of Life (QOL): Reflecting on patients’ wishes and considering how to support them.” Classes were conducted face-to-face in 2019 and remotely between 2021 and 2023; each class was conducted in June. In 2019, classes were conducted over two days (half a day each). From 2021 to 2023, classes were conducted over 3 days (half a day each). The main additions to the distance classes were a review of Assembly I and Assembly II and an explanation of network literacy for distance classes. The content of the classes changed slightly each year, as it was refined based on discussions among faculty members (Table 1). The Team-Based Learning method was used each year^8–10^; it consisted of an individual readiness assurance test (iRAT) to check readiness, a team test (tRAT: team readiness assurance test), an appeal, feedback, application activities, and peer evaluation. Orientation sessions were held in each department approximately 2 weeks before the classes, and the preparation materials were distributed.

### Survey methods and contents

Students were surveyed before and after class using the Japanese version of the Readiness for Interprofessional Learning Scale (RIPLS).^11^ The Cronbach’s alpha for this scale was 0.74. The RIPLS consists of 19 items in three subscales: 13 items for “Teamwork and collaboration,” 2 items for “IPE opportunities,” and 4 items for “Uniqueness of profession.” Each item was measured on a 5-point Likert scale ranging from 1: “Strongly Disagree” to 5: “Strongly Agree.”

Teachers were also asked, ‘Overall, which do you think is preferable: face-to-face learning or distance learning?’ The possible responses were “face-to-face learning,” “distance learning,” or “Neither.” Furthermore, they were asked to provide free-form responses regarding the advantages and disadvantages of face-to-face and distance learning through interprofessional education.

### Analysis

Students were surveyed using the Japanese version of the RIPLS. Total scores for “Teamwork and collaboration,” “IPE opportunities,” “Uniqueness of professional,” and “Overall” before and after the interprofessional education program were compared using the Wilcoxon signed-rank test. Furthermore, the difference in scores before and after face-to-face learning in 2019 and the difference in scores for each year before and after distance learning were compared using the Mann–Whitney U test. The statistical software used was SPSS ver. 29.0 (IBM Corporation), and the significance level was set at p<5%. Multiple comparisons were performed using the Bonferroni method.^12,13^

Free form responses from teacher surveys were coded and categorized based on their similarities and differences. The results were repeatedly reviewed by researchers to ensure their validity.

### Ethical considerations

The purpose, methods, and content of the study were explained to the participants in writing. They were informed that participation was voluntary and that refusal would not result in any disadvantages. Students were asked to enter their student ID numbers for comparison before and after classes; however, new numbers were assigned to the data obtained from the students, and student ID numbers were deleted to ensure anonymity. The teacher surveys were conducted anonymously. Submission of responses constituted consent for the study. This study was approved by the Fujita Health University Medical Research Ethics Review Committee (HM18-250).

## Results

In 2019, of the 875 eligible students, 837 completed the pre- and post-class surveys (with a response rate of 95.7%). The response rates for 2021, 2022, and 2023 were 74.8%, 80.1%, and 65.2%, respectively. The valid response rate was 100% for all the years. Table 2 presents the student attributes. Students from the pharmacy, Medicine, and Nursing faculties participated most frequently, accounting for 9.7%–29.2% of the total.

Of the 83 teachers, 41 responded (with a response rate of 49.4%), resulting in a valid response rate of 100%. Table 3 presents the attributes of the teachers. Teachers in the Department of Health Sciences accounted for the largest proportion (26.8 %).

### Comparison of scores between face-to-face and distance learning

In all academic years, there was a significant increase in overall scores of the RIPLS and the subscale “Teamwork and collaboration” after classes compared to that before classes (Table 4). A significant increase in “IPE opportunities” was observed in face-to-face learning, but no significant difference was observed in distance learning. The difference in “Uniqueness of profession” before and after classes ranged from −0.1 to 0.2 points, reaching ~0 points in each year. A significant difference in “Uniqueness of profession” was detected in face-to-face learning in 2019, but no significant difference was observed in distance learning from 2021 to 2023.

Comparing the differences in scores before and after the classes between face-to-face and distance learning, the overall score increase was significantly larger for face-to-face learning than for distance learning (Table 5). For “teamwork and collaboration,” the increase in scores was smaller for distance learning than for face-to-face learning in 2021 and 2022; however, no significant difference was observed between distance learning and face-to-face learning in 2023. For “IPE opportunities,” the increase in scores was significantly larger for face-to-face learning than for distance learning. For “Uniqueness of profession,” the increase in scores for distance learning in 2021 was significantly smaller than for face-to-face learning; however, no significant difference was found between face-to-face and distance learning in 2022 and 2023.

When teachers were asked, “Overall, which do you think is preferable: face-to-face or distance learning?” 70.7% answered “face-to-face learning,” 4.9% answered “distance learning,” and 24.4% answered “Neither” with “face-to-face learning” being the most common answer (Table 6).

### Advantages and disadvantages of face-to-face and distance learning

The advantages of face-to-face learning through interprofessional education as perceived by teachers consisted of five categories and ten subcategories, whereas the disadvantages of face-to-face learning consisted of six categories and nine subcategories. The advantages of distance learning consisted of five categories and twelve subcategories, whereas the disadvantages of face-to-face learning consisted of five categories and six subcategories (Table 7 and Supplementary Tables 1–4). The categories are indicated using [ ].

The advantages of face-to-face learning were summarized as [smooth communication and discussion], [improved concentration and sense of participation], [sense of team unity], [stimulation from other teams], and [easy observation, exchange of information, and support from teachers].

The disadvantages of face-to-face learning were [time-consuming and costly to move], [difficulty in securing classrooms that can accommodate large numbers of people], [heavy burden of preparation and administration], [difficulty in listening to discussion content due to noise from other teams], [negative impact when members with low awareness of participation are in view], and [difficulty for teachers to grasp and evaluate discussion content].

Contrarily, the advantages of distance learning were summarized as follows: [ease of participation due to no need for movement, cost, or location], [less burden of preparation and administration], [focus on intra-team discussions]; [teachers can monitor team discussions and evaluate appropriately], and [no risk of infection].

The disadvantages of distance learning were: [less smooth communication and discussion], [difficulty in maintaining concentration], [difficulty in grasping the situations of other teams], [difficulty for teachers to intervene], and [difficulty in dealing with communication failures].

## Discussion

### Comparison of scores between face-to-face and distance learning

In all years, RIPLS overall scores and scores on the subscale “Teamwork and collaboration” significantly increased after classes compared to before classes. This suggests that our interprofessional education program was effective in enhancing teamwork and collaboration among participating students in both face-to-face and distance learning. The TBL method adopted in this study emphasizes teamwork,^14^ and the possibilities that “teamwork and collaboration” scores can increase through team activities.

In face-to-face learning, the IPE opportunity scores significantly increased after class. The difference in “Expertise” scores was nearly zero points before and after classes every year. Although a significant difference in “Uniqueness of profession” was detected in face-to-face learning, the difference was so small (0.2 points) that it may not be significant. In face-to-face interprofessional education for medical, dental, and pharmaceutical students at another Japanese university, “IPE opportunities” and “Uniqueness of profession” scores did not increase after classes.^12^ This survey was conducted among three departments at the same university, whereas ours was a large-scale interprofessional education program across seven departments at four universities. This may have contributed to the increased “IPE opportunities” seen in face-to-face learning. Regarding “Uniqueness of profession,” our results suggest that students did not feel that their expertise increased in either face-to-face or distance learning. This may be because this interprofessional education program was not conducted in a clinical setting. We believe that the program needs to be modified to enable students increase their expertise.

When comparing the changes in RIPLS scores before and after the program between face-to-face and distance learning, the increase in overall scores was significantly greater for face-to-face learning than for distance learning. Furthermore, given that a higher percentage of teachers responded that “face-to-face learning is better,” we believe that, overall, face-to-face learning may be more effective in interprofessional education. The change in the overall pre- and post-class total scores for “Teamwork and collaboration” was significantly smaller in 2021 and 2022 for distance learning than for face-to-face learning; however no significant difference was observed in 2023. The distance learning courses conducted from 2021 to 2023 were revised annually based on discussions among teachers; therefore, the content was not identical. As a result of these refinements, the 2023 distance learning courses may have resulted in students experiencing improvements in teamwork and collaboration, similar to face-to-face learning. Furthermore, students may have gained experience in distance learning and discussions on other subjects during the COVID-19 pandemic, influencing their results.

This study also revealed the benefits of distance learning. By further refining the content to strengthen faculty involvement and promote peer learning within teams, “teamwork and collaboration” may achieve educational outcomes equivalent to those of in-person instruction. According to the Center for the Advancement of Interprofessional Education,^15^ “IPE occurs when two or more professionals learn with, from, and about each other to improve collaboration and quality of care.” Therefore, interactive communication among team members is essential for teamwork.^16^ While some areas detected that “Uniqueness of profession” showed significantly higher score increases in face-to-face learning than in distance learning, the actual increase in scores was extremely small. Therefore, we could not conclude whether face-to-face learning enhanced expertise.

Educational effectiveness was assessed using the Japanese version of the RIPLS. The developers of the scale reported a Cronbach’s alpha of 0.74, indicating a certain degree of reliability. Factor analysis revealed that the scale is composed of three subscales: 13 items for “teamwork and collaboration,” 2 items for “IPE opportunities,” and 4 items for “Uniqueness of profession” (Tamura, 2012). However, subsequent research has indicated that the scale’s reliability and validity are not as expected by the developers. In a study of students from three departments who received interprofessional education,^17^ the Japanese version of the RIPLS had low Cronbach’s alpha for the subscale “Uniqueness of profession” and “IPE opportunities” and “Uniqueness of profession” were not independent constructs, resulting in problems with construct validity. It has also been reported previously that other language versions of the RIPLS have low reliability for some subscales, construct validity may not be achieved as proposed by the developers, and that ceiling effects exist.^18–20^ As other indicators for evaluating the effectiveness of interprofessional education, such as the Chiba Interprofessional Competency Scale,^21^ which was developed to measure interprofessional competence, may be effective, their use should be considered in future research.

### Advantages and disadvantages of face-to-face and distance learning

The advantages of face-to-face classes were categorized as “smooth communication and discussion,” “improved concentration and sense of participation,” “sense of team unity,” “stimulation from other teams,” and “easy observation, exchange of information, and support from teachers.” These findings suggest that face-to-face learning encourages focused communication and discussion within teams, with a high sense of participation. Students also likely progressed with inspiration from other teams and support from their instructors. In contrast, distance learning presents communication challenges, such as “less smooth communication and discussion,” “difficulty in grasping the situations of other teams,” and “difficulty for teachers to intervene.” This likely explains why many instructors reported that face-to-face learning was more effective than distance learning. Previous studies reported that communication is often insufficient in distance learning.^22^ Since mutual discussion and interaction among participants are important in interprofessional education, communication issues must be considered when implementing interprofessional education via distance learning. Contrarily, distance learning had the following advantages: [ease of participation due to no need for movement, cost, or location], [less burden of preparation and administration], [focus on intra-team discussions], [teachers can monitor team discussions and evaluate appropriately], and [no risk of infection]. Therefore, it is believed that there is potential for educational effectiveness to be achieved by connecting universities that are far apart, with teachers guiding team discussions and repeatedly evaluating and improving them. In the future, it will be important to understand the advantages and disadvantages of both face-to-face and distance learning, and to create interprofessional education classes that make the most of these advantages.

### Limitations of the study

The questionnaire survey in this study was based on self-assessment; therefore, there are undeniable issues with objectivity. However, since the same students conducted the evaluations before and after the class, we believe that this did not affect the pre-post comparison.

Face-to-face learning was conducted over two days, whereas remote classes spanned three days. A longer duration of distance learning is necessary to include instruction on network literacy. Since all years used the “QOL” theme and TBL methodology, we considered the impact on comparability to be minimal.

In this survey, the low response rate of students in remote classes (65.2% to 80.1%) suggested a potential selection bias. The response rate from teachers was only about half, indicating that the advantages and disadvantages of face-to-face and distance learning may not have been fully captured.

This study compared the results before and after the Assembly III class, and the possibility that experiences in other classes or activities influenced the results cannot be ruled out.

## Conclusions

- In all years, RIPLS overall scores increased significantly compared to before classes, as scores on the “teamwork and collaboration” subscale, suggesting that both face-to-face and distance learning were effective in enhancing student teamwork and collaboration.

- Since “IPE opportunities” significantly increased in face-to-face learning, it is possible that students perceived increased opportunities for IPE in our large-scale, face-to-face, interprofessional education.

- Since “Uniqueness of profession” showed little change before and after classes in both face-to-face and distance learning, measures to enhance expertise are necessary in both face-to-face and distance learning.

- The increase in RIPLS overall scores before and after classes was significantly larger in face-to-face learning than distance learning, and a higher percentage of teachers responded that “face-to-face learning is better,” suggesting that face-to-face learning may be more effective overall.

- The change in scores for “Teamwork and collaboration” was significantly smaller in distance learning in 2021 and 2022 than in face-to-face learning, but no significant difference was observed between distance learning in 2023 and face-to-face learning. This suggests that further refinement of distance learning may achieve the same educational effectiveness in “Teamwork and collaboration” as face-to-face learning.

- The advantages teachers see in face-to-face learning is [smooth communication and discussion], [improved concentration and sense of participation], [sense of team unity], [stimulation from other teams], and [easy observation, exchange of information, and support from teachers]. The disadvantages include: [time-consuming and costly to move], [difficulty in securing classrooms that can accommodate large numbers of people], [heavy burden of preparation and administration], [difficulty in hearing discussion content due to noise from other teams], [negative impact when members with low awareness of participation are in view], and [difficulty for teachers to grasp and evaluate discussion content].

- Distance classes had the following advantages: [ease of participation due to no need for movement, cost, or location], [less burden of preparation and administration], [focus on intra-team discussions]; [teachers can monitor team discussions and evaluate appropriately], and [no risk of infection]. However, there are also disadvantages such as [less smooth communication and discussion], [difficulty in maintaining concentration], [difficulty in grasping the situations of other teams], [difficulty for teachers to intervene], and [difficulty in dealing with communication failures].

## Figures and Tables

**Table 1:** Primary learning structure and contents for each year in interprofessional education

Face-to-face learning in 2019	Day 1	iRAT·tRAT (type of occupation), appeals, feedback, application activities “How do patients wish to live?” and “Thinkning from the perspective of the patients and their family about what can be done to help them realize the way they wish to live.”, Presentations and discussions
	Day 2	Application activities “Thinking about support for patients who have relapsed” and “Thinking about the role of the professional you aspire to be”, presentation and discussion, peer evaluation
	Day 3	None
Distance learning in 2021	Day 1	Review of Assembly I and II, network literacy
	Day 2	iRAT·tRAT (type of occupation), appeals, feedback, application activities “How do patients wish to live?” and “Thinkning from the perspective of the patients and their family about what can be done to help them realize the way they wish to live.”, Presentations and discussions
	Day 3	iRAT·tRAT (QOL, ACP: Advance care planning), appeals, feedback, application activities “Imagining the feels of a patient who has relapsed and thinking about what you can do to alleviate their anxiety.”, peer evaluation
Distance learning in 2022	Day1	Network literacy, iRAT·tRAT (type of occupation, QOL), appeals, feedback, application activities “Thinking about how you would feel if you were a patient or family member”, presentation and discussion
	Day2	iRAT·tRAT (ACP, COVID-19), appeals, feedback, application activities “Thinking from the perspective of those around the patient”, “Considering the feelings of the patient who hase been undergoing treatment.”
	Day3	Presentation and discussion, peer evaluation
Distance learning in 2023	Day1	Network literacy, iRAT·tRAT (type of occupation), appeals, feedback, application activities “Thinking about how you would feel if you were a patient or family member”, and “Thinking from the perspective of those around the patient”, presentation and discussion
	Day2	iRAT·tRAT (QOL, ACP), appeals, feedback, application activities “Considering the feelings of the patient who hase been undergoing treatment.”, and “Thinking about what each profession can do for patients, their families, and those around them”
	Day3	Presentation and discussion, peer evaluation

**Table 2:** Attributes of the students

Year	2019				2021				2022				2023		
Department	Faculty and grade	n	%		Faculty and grade	n	%		Faculty and grade	n	%		Faculty and grade	n	%
Medicine	Medicine, grade3	107	12.8		Medicine, grade3	75	9.7		Medicine, grade3	100	13.1		Medicine, grade3	90	13.5
Medical Sciences	Medical Technology, grade 3	96	11.5		Medical Technology, grade 3 (including Medical Technology and Clinical Engineering programs)	102	13.1		Medical Technology, grade 3 (including Medical Technology and Clinical Engineering programs)	101	13.1		Medical Technology, grade 3 (including Medical Technology and Clinical Engineering programs)	90	13.5
	Clinical Engineering, grade 3	47	5.6												
	Radiological Technology, grade 3	59	7.0		Radiological Technology, grade 3	71	9.1		Radiological Technology, grade 3	66	8.6		Radiological Technology, grade 3	60	9.0
	Medical Management and Information Science, grade 4	32	3.8		Medical Management and Information Science, grade 4	33	4.2								
Health Sciences	Nursing, grade 3	136	16.2		Nursing, grade 3	99	12.7		Nursing, grade 3	108	14.1		Nursing, grade 3	80	12.0
	Rehabilitation, grade 3	52	6.2		Rehabilitation, grade 3	90	11.6		Rehabilitation, grade 3	83	10.9		Rehabilitation, grade 3	76	11.5
Pharmacy	Pharmacy, grade 4	244	29.2		Pharmacy, grade 4	203	26.1		Pharmacy, grade 4	207	27.0		Pharmacy, grade 4	181	27.2
Social Welfare	Social Welfare, grades 3 and 4	34	4.1		Social Welfare, grades 3 and 4	8	1.0		Social Welfare, grades 3 and 4	26	3.4		Social Welfare, grade 3	8	1.2
Dentistry	Dentistry, grade 6	22	2.6		Dentistry, grade 3	91	11.7		Dentistry, grade 3	66	8.6		Dentistry, grade 3	74	11.1
Psychological and Physical Science	Health Nutrition, grade 4	8	1.0		Health Nutrition, grade 4	5	0.6		Health Nutrition, grade 4	9	1.2		Health Nutrition, grade 4	7	1.1
	Total	837	100		Total	777	100		Total	766	100		Total	666	100

**Table 3:** Attributes of the teachers

Department	n	%
Medicine	4	9.7
Medical Sciences	9	22.0
Health Sciences	11	26.8
Pharmacy	9	22.0
Social Welfare	2	4.9
Dentistry	5	12.2
Psychological and Physical Science	1	2.4
Total	41	100.0

**Table 4:** Comparison of RIPLS scores before and after class

		Face-to-face learning in 2019 (n=837)	Distance learning in 2021 (n=777)	Distance learning in 2022 (n=766)	Distance learning in 2023 (n=666)
		Mean±SD	Mean±SD	Mean±SD	Mean±SD
Teamwork and collaboration (13 items)	Before class	50.4±5.9	52.4±5.8	51.8±6.2	52.1±6.5
	After class	52.9±6.2	53.6±6.6	53.1±6.4	54.1±6.6
	Differences in scores before and after class^1^	2.5	1.2	1.3	2.0
	p value	<0.001	<0.001	<0.001	<0.001
IPE opportunities (2 items)	Before class	6.9±2.0	7.8±1.9	7.6±2.0	7.5±2.0
	After class	7.3±2.2	7.7±2.1	7.5±2.2	7.5±2.3
	Differences in scores before and after class^1^	0.4	–0.1	–0.1	0.0
	p value	<0.001	0.861	0.484	0.725
Uniqueness of profession (4 items)	Before class	12.4±2.0	13.0±1.8	12.8±2.0	12.9±2.0
	After class	12.6±2.1	12.9±2.1	12.8±2.1	12.9±2.3
	Differences in scores before and after class^1^	0.2	–0.1	0.0	0.0
	p value	0.003	0.444	0.524	0.956
Overall (19 items)	Before class	69.7±7.5	73.1±7.5	72.1±7.8	72.5±8.3
	After class	72.8±7.9	74.2±8.5	73.4±8.1	74.5±8.6
	Differences in scores before and after class^1^	3.1	1.1	1.3	2.0
	p value	<0.001	<0.001	<0.001	<0.001

Wilcoxon signed rank test (significant difference at p<0.05/16=0.003125 for multiplicity with Bonferroni correction) SD, standard deviation ^1^ “after class scores” minus “before class scores”

**Table 5:** Comparison of scores before and after class differences between 2019 face-to-face classes and distance learning of other years

	Face-to-face learning in 2019 (n=837)		Distance learning in 2021 (n=777)	Comparison between 2019 and 2021		Distance learning in 2022 (n=766)	Comparison between 2019 and 2022		Distance learning in 2023 (n=666)	Comparison between 2019 and 2023
	Difference in scores before and after class^1^		Difference in scores before and after class^1^	p value		Difference in scores before and after class^1^	p value		Difference in scores before and after class^1^	p value
Teamwork and collaboration (13 items)	2.5		1.2	<0.001		1.3	<0.001		2.0	0.095
IPE opportunities (2 items)	0.4		–0.1	<0.001		–0.1	<0.001		0.0	<0.001
Uniqueness of profession (4 items)	0.2		–0.1	0.004		0.0	0.104		0.0	0.068
Overall (19 items)	3.1		1.1	<0.001		1.3	<0.001		2.0	0.001

Mann–Whitney test (significant difference at p<0.05/12=0.004166 for multiplicity with Bonferroni correction) ^1^ “after class scores” minus “before class scores”

**Table 6:** Ratio on whether face-to-face or distance learning is preferable

	n	%
Face-to-face learning	29	70.7
Distance learning	2	4.9
Neither	10	24.4
Total	41	100

**Table 7:** Advantages and disadvantages of face-to-face and distance learning (categories)

	Advantages	Disadvantages
Face-to-face learning	Smooth communication and discussion Improved concentration and sense of participation Sense of team unity Stimulation from other teams Easy observation, exchange of information, and support from teachers	Time-consuming and costly to move Difficulty in securing classrooms that can accommodate large numbers of people Heavy burden of preparation and administration Difficulty in listening to discussion content due to noise from other teams Negative impact when members with low awareness of participation are in view Difficulty for teachers to grasp and evaluate discussion content
Distance learning	Ease of participation due to no need for movement, cost, or location Less burden of preparation and administration Focus on intra-team discussions Teachers can monitor team discussions and evaluate appropriately No risk of infection	Less smooth communication and discussion Difficulty in maintaining concentration Difficulty in grasping the situations of other teams Difficulty for teachers to intervene Difficulty in dealing with communication failures

## Supplementary Material

Supplementary Tables

PDF-Japanese
