# Supplementary material for: Interprofessional education at four joint medical and welfare universities: A comparison of face-to-face and distance learning
Source: Fujita Med J. 2026 Feb 28;12(2):172–8. doi: 10.20407/fmj.2025-030 (PMC13129710; doi:10.20407/fmj.2025-030)
Supplement: Supplementary file 1 — Supplementary Tables [file fmj-12-172_s1.pdf]

Supplementary Table S1　Advantages of face-to-face learning

Categories	Subcategories
Smooth communication and discussion	Easy team discussion
	Smooth communication
Improved concentration and sense of participation	Students can concentrate on their work
	Increased student participation
	No free riders
Sense of team unity	Sense of team unity
Stimulation from other teams	Stimulation from other teams
Easy observation, exchange of information, and support by teachers	Teachers can easily observe the overall situation
	Easier for teachers to reach out to and support students
	Easy sharing and exchange of information among teachers

Supplementary Table S2　Disadvantages of face-to-face learning

Categories	Subcategories
Time-consuming and costly to move	Moving time is long
	Need money to move
Difficult to secure a classroom that can accommodate large numbers of people	Difficult to secure a classroom that can accommodate large numbers of people
Heavy burden of preparation and administration	Heavy burden of preparation and administration
Difficult to hear discussion content through other teams' voices	Difficult to hear discussion content through other teams' voices
Negative impact when people with low awareness of participation are in view	Negative impact on visibility of students with low participatio
	Negative impact on students when they see faculty members with low awareness
Difficult for teachers to grasp and evaluate discussion content	Teachers have difficulty keeping track of students' discussions
	Difficult to evaluate faculty due to difficulty in hearing student conversations

Supplementary Table S3　Advantages of distance learning

Categories	Subcategories
Easier to participate due to no need for movement, cost, or location	No need to move
	No cost burden
	No need to secure a location
	Minimal timetable adjustments and easy participation
Less burden of preparation and administration	Less burden of preparation and administration
Focus on intra-team discussions	Focus on intra-team discussions
	Less private language unrelated to the discussion
	Less concentration of work on specific students
Teachers can monitor team discussions and evaluate appropriately	Easy for teachers to keep track of team discussions and materials
	Teachers can easily see each team's activities, making evaluations more relevant
	Teachers are responsible for the activities
No risk of infection	No risk of infection

Supplementary Table S4 Disadvantages of distance learning

Categories	Subcategories
Communication and discussion are not smooth	Difficulty in facilitating discussion
	Communication is difficult
Difficult to stay focused	Difficult to stay focused
Difficult to grasp the situation of other teams	Difficult to grasp the situation of other teams
Difficult for teachers to intervene	Difficult for teachers to intervene
Difficult to respond to communication failures	Difficult to respond to communication failures
