# Supplementary material for: Interprofessional education at four joint medical and welfare universities: A comparison of face-to-face and distance learning
Source: Fujita Med J. 2026 Feb 28;12(2):172–8. doi: 10.20407/fmj.2025-030 (PMC13129710; doi:10.20407/fmj.2025-030)
Supplement: Supplementary file 2 — PDF-Japanese [file fmj-12-172_s2.pdf]

Title:

医療・福祉系 4 大学合同の多職種連携教育―対面授業と遠隔授業の比較―

Interprofessional education at four joint medical and welfare universities: A comparison of face-to-face and distance learning

Running title:

多職種連携教育における対面授業と遠隔授業の比較

Comparison of face-to-face and distance learning in interprofessional education

Authors:

Sayuri Nakamura, RN, PHN, PhD[1], Atsuhiko Ota, MD, PhD, MPH[2], Eizou Umezawa, PhD[3], Mihoko Itoh, OTR, PhD[4], Miho Miyamoto, PHN, RN, PhD[1], Yuki Higashimoto, MT, PhD[5], Fumiaki Horiba,PhD[6], Hiroyuki Kamei, PhD[7], Shigetaka Tomoda, DDS, PhD[8], Masatsugu Ohtsuki, MD PhD[9]

[1]Faculty of Nursing, School of Health Sciences, Fujita Health University, Toyoake, Aichi, Japan

[2]Department of Public Health, School of Medicine, Fujita Health University, Toyoake, Aichi, Japan

[3]Department of Medical Sciences Education, School of Medical Sciences, Fujita Health University, Toyoake, Aichi, Japan

[4]Faculty of Rehabilitation, School of Health Sciences, Fujita Health University, Toyoake, Aichi, Japan

[5]Department of Clinical Microbiology, School of Medical Sciences, Fujita Health University, Toyoake, Aichi, Japan

[6]Department of Fundamental Education, School of Medical Sciences, Fujita Health University, Toyoake, Aichi, Japan

7Faculty of Pharmacy, Meijo University, Nagoya, Aichi, Japan

[8]Department of Operative Dentistry, School of Dentistry, Aichi-Gakuin University, Nagoya, Aichi, Japan

[9]Department of Clinical General Medicine, School of Medicine, Fujita Health University, Toyoake, Aichi, Japan

Manuscript Type:

Original Article

Corresponding author:

Sayuri Nakamura, RN, PHN, PhD

Faculty of Nursing, Fujita Health University School of Health Sciences, 1-98, Dengakugakubo, Kutsukake-cho, Toyoake, Aichi, 470-1192, Japan

Tel No: +81-562-93-9077

E-mail: sayuri@fujita-hu.ac.jp

要旨
目的：対面授業と遠隔授業の多職種連携教育の効果を比較することを目的とした。
方法：多職種連携教育に参加した医療・福祉系4大学の学生を対象としてRIPLS日本語版を用いて調査し、対面授業と遠隔授業を比較した。また、対面授業と遠隔授業の両方に参加した教員を対象に対面授業と遠隔授業のメリットとデメリットを自由記述にて調査し、コード化・カテゴリー化した。
結果：対面・遠隔共に授業後のRIPLS全体合計と「チームワークとコラボレーション」の得点が有意に上昇した。「IPEの機会」は対面授業で有意な上昇を認めた。「専門性」は対面・遠隔共に授業前後でほとんど変化しなかった。授業前後の全体合計の得点差は、対面の方が遠隔よりも有意に大きかった。対面授業のメリットは、【コミュニケーション・議論が円滑】等であった。遠隔授業は距離が離れた大学を繋いで授業を行うのに有用だったが、【コミュニケーション・議論が円滑でない】【集中力が保ちにくい】というデメリットが存在した。
結論：遠隔よりも対面授業の方が効果は高い可能性が示唆された。「IPEの機会」は遠隔授業で、「専門性」は対面・遠隔授業共に課題があることが示唆された。
キーワード：多職種連携教育、対面授業、遠隔授業、教育効果、チーム基盤型学習

はじめに

医療・福祉の現場は複雑で多様な問題を抱えており、専門性を超えて連携する必要があり、卒前の効果的な多職種連携教育が求められている[1,2]。

藤田医科大学は3学部5学科を有する総合医療大学であり、1971年の大学設立時より学部・学科混成のアセンブリ教育を実施している。これは多職種連携教育（Interprofessional education：IPE）であり、1年生ではアセンブリⅠ、2年生ではアセンブリⅡ、3年生ではアセンブリⅢ、4年生ではアセンブリⅣを行っている（アセンブリⅠ～Ⅲは必修、Ⅳは選択科目）。身につける力は、アセンブリⅠでは「コミュニケーション」、アセンブリⅡでは「チームワーク」、アセンブリⅢでは「患者・利用者・家族・コミュニティ中心の考え方」と「職種の理解」であり、アセンブリⅣでは「協働の実践」である。

今回はアセンブリⅢに焦点をあてる。今までの研究において、アセンブリⅢの授業前に比べて授業後に多職種連携教育の得点が有意に上昇したこと[3,4]、グループワークの参加の仕方とピア評価には有意な関連があること[5]を報告した。2019年までのアセンブリⅢは全て対面授業にて実施した。その後、コロナウイルス感染症のパンデミックにより、従来の対面授業が制限されたため、2020年から2023年までは遠隔授業で多職種連携教育を行った。先行研究において、4学部合同による多職種連携教育の対面と遠隔授業では、評価による違いはなかったという報告がある[6]。看護学部の学生を対象とした多職種連携教育の対面と遠隔授業を行った研究では、対面授業よりも遠隔授業の方が評価得点は高かったという報告があり[7]、結果に違いがみられている。今回我々が行った医療・福祉系4大学7学部合同による多職種連携教育において、対面授業と遠隔授業を比較し、教育効果を確認する必要があると考えている。研究成果は、多職種連携教育の授業を改善する資料となると考える。

そこで、本研究では対面授業と遠隔授業の多職種連携教育の効果を比較することを目的とした。

研究方法

対象

2019 年度～2023 年度の多職種連携教育に参加した医療・福祉系 4 大学 7 学部の学生を対象とし、授業前・後ともにアンケート調査に回答した者を解析対象とした。2020 年度はコロナウイルス感染症の影響で藤田医科大学の学生のみでの実施となったため、解析対象から除いた。対象とした 7 学部は、医学部、医療科学部、保健衛生学部、薬学部、社会福祉学部、歯学部、心身科学部である。

また、多職種連携教育の対面授業と遠隔授業の両方に参加した経験のある教員を対象とした。

多職種連携教育

2019 年度から 2023 年度の授業のテーマは、いずれも「QOL（Quality of Life）:患者のどう生きたいかという願いに思いをめぐらせ、患者の願いにどう寄り添うかを考える」であった。2019 年度は対面授業で、2021 年度～2023 年度は遠隔授業にて実施した。時期はいずれも 6 月であった。2019 年度は 2 日間（半日ずつ）で実施した。2021 年度～2023 年度は 3 日間（半日ずつ）であった。遠隔授業の方が 1 日多かったが、遠隔授業に追加した主な部分は、アセンブリⅠとⅡの復習と遠隔授業に伴うネットワークリテラシーの説明であった。教員間の議論に基づいてブラッシュアップしたため、授業内容は毎年少しずつ変化した（表 1）。いずれの年度も教育手法は、TBL（Team-based Learning：チーム基盤型学習）とした[8-10]。授業の約 2 週間前に各学科でオリエンテーションを行い、予習資料を渡した。いずれの年度も準備確認の個人テスト iRAT （Individual readiness assurance test）、チームテスト（tRAT：Team readiness assurance test）、アピール、フィードバック、応用課題、ピア評価からなる TBL に沿った構成で行われた。

調査方法・内容

授業前と授業後に RIPLS（Readiness for Interprofessional Learning Scale）日本語版[11]を用いて学生を対象にアンケート調査を行った。この尺度における Cronbach’s α係数は 0.74 である。RIPLS は、「チームワークとコラボレーション」13 項目、「IPE の機会」2 項目、「専門性」4 項目の３つの下位尺度 19 項目から構成されている。各項目は「1 点：全くそう思わない」～「5 点：きわめてそう思う」の５段階リッカート尺度にて測定した。

また、教員を対象に「総合的にみて、対面授業と遠隔授業のどちらが望ましいと考えるか」を尋ね、「対面授業」「遠隔授業」「どちらともいえない」から回答を選んでもらった。さらに、多職種連携教育による対面授業のメリットとデメリット、および遠隔授業のメリットとデメリットを自由記述にて回答してもらった。

分析

学生に調査した RIPLS 日本語版において、「チームワークとコラボレーション」「IPE の機会」

「専門性」「19 項目全体」の合計得点を多職種連携教育の前後を Wilcoxon の符号付順位検定にて比較した。さらに、2019 年対面授業前後の得点差と遠隔授業前後における各年の得点差を Mann-whitney U 検定にて比較した。統計ソフトは SPSSver29.0(IBM 株式会社)を使用し、有意水準は 5%未満とした。多重比較を行った際はボンフェローニ法による調整を行った[12,13]。

教員に調査した自由記述の内容はコード化し、類似性・相違性に基づいてカテゴリー化した。研究者間で繰り返し検討し、妥当性の確保に努めた。

倫理的配慮

研究対象者に文書を用いて研究の目的、方法、内容の説明を行った。研究は自由意思で行われるものであり、断ることによって不利益を被ることは一切ないことを説明した。授業前後の比較をするために学籍番号を入力してもらったが、学生から得られたデータには新たな番号を付し、学籍番号を削除して個人が特定できないようにした。教員に対する調査は無記名で行った。回答の提出をもって研究の同意をされたとした。本研究は、藤田医科大学医学研究科倫理審査委員会の承認を得て実施した (HM18-250)。

結果

2019 年度は対象学生 875 名のうち、授業前・後ともに回答が得られたのが 837 名(回収率 95.7%)であった。2021 年度～2023 年度の回収率はそれぞれ 74.8%、80.1%、65.2%であった。有効回答率は全ての年度において 100%であった。対象学生の属性を表 2 に示す。薬学科、医学科、看護学科の学生の参加が 9.7～29.2%と多かった。

教員は 83 名中 41 名より回答が得られ (回収率 49.4%)、有効回答率 100%であった。教員の属性を表 3 に示す。保健衛生学部の教員が 26.8%と最も多かった。

対面授業と遠隔授業の得点の比較

全ての年度において授業前に比べて授業後の RIPLS 全体合計得点および下位尺度「チームワークとコラボレーション」の得点が有意に上昇した (表 4)。「IPE の機会」は対面授業で有意な上昇を認めたが、遠隔授業では有意差を認めなかった。「専門性」の授業前後の差は-0.1～0.2 点であり、いずれの年もほぼ 0 点を示した。2019 年度対面授業で「専門性」に統計学的有意差が検出されたが、2021 年～2023 年の遠隔授業では有意差を認めなかった。

授業前後の得点差を対面授業と遠隔授業で比較したところ、全体合計得点は、対面授業の方が遠隔授業よりも得点上昇が有意に大きかった (表 5)。「チームワークとコラボレーション」は、2021 年・2022 年遠隔授業における得点上昇は対面授業よりも小さかったが、2023 年遠隔授業と対面授業では有意差を認めなかった。「IPE の機会」は対面授業の方が遠隔授業よりも得点上昇が有意に大きかった。「専門性」は、2021 年遠隔授業における得点上昇は対面授業よりも有意に小さかったが、2022・2023 年遠隔授業と対面授業の間では有意差を認めなかった。

「総合的に対面授業と遠隔授業ではどちらが望ましいと考えるか」の教員への質問に対して、「対面授業」と回答した者の割合は 70.7%、「遠隔授業」は 4.9%、「どちらともいえない」は 24.4%

であり、「対面授業」と答えた者が最も多かった（表 6）。

対面・遠隔授業のメリット・デメリット

教員が考える多職種連携教育による対面授業のメリットは、5 カテゴリー、10 サブカテゴリーから構成され、対面授業のデメリットは、6 カテゴリー、9 サブカテゴリーから構成された。遠隔授業のメリットは、5 カテゴリー、12 サブカテゴリーから構成され、対面授業のデメリットは、5 カテゴリー、6 サブカテゴリーから構成された（表 7 および付表 1～4）。以下、カテゴリーを【 】で示す。

対面授業のメリットは、【コミュニケーション・議論が円滑】【集中力・参加意識の向上】【チームの一体感】【他のチームからの刺激】【教員による観察・情報交換・支援が容易】に集約された。

対面授業のデメリットは、【移動に時間・コストがかかる】【大人数収容可能な教室の確保が困難】【準備・運営の負担が大きい】【他のチームの音で討論内容が聞こえづらい】【参加意識の低い人が視界に入ると悪影響】【教員が討論内容を把握しづらく評価が困難】であった。

一方、遠隔授業のメリットは、【移動・コスト・場所の確保が不要なために参加しやすい】【準備・運営の負担が小さい】【チーム内討論に集中】【教員がチーム内の議論の状況を把握でき、評価が適切】【感染リスクがない】に集約された。

遠隔授業のデメリットは、【コミュニケーション・議論が円滑でない】【集中力が保ちにくい】【他のチームの状況を把握しづらい】【教員が介入しづらい】【通信トラブル時の対応が困難】であった。

考察

対面授業と遠隔授業の得点の比較

全ての年度で授業前に比べて授業後に RIPLS 全体合計得点と下位尺度「チームワークとコラボレーション」の得点が有意に上昇した。このことから、我々が行った多職種連携教育は、対面授業でも遠隔授業でも参加学生のチームワークとコラボレーションを高める効果があったと考える。今回取り入れた TBL の手法はチームで協力し合うことを重要視しており [14]、チーム活動を通して「チームワークとコラボレーション」の得点が上昇した可能性がある。

「IPE の機会」は対面授業で授業後の得点が有意に上昇した。「専門性」の授業前後差はいずれの年度もほぼ 0 点であった。対面授業でのみ「専門性」の有意差が検出されたものの、0.2 点と極わずかな値であるため、意味のある差ではない可能性がある。日本の他の大学で医学部・歯学部・薬学部学生を対象とした対面による多職種連携教育において、「IPE の機会」と「専門性」の得点が授業後に伸びなかったという研究報告がある [12]。これは同一大学内の 3 学部での調査であったのに対し、我々が行った授業は 4 大学 7 学部でより大規模な多職種連携教育であったことが関係して「IPE の機会」が対面授業で上昇したのかもしれない。「専門性」に関しては、我々の授業においても対面のみならず遠隔でも学生達は専門性が高まったと感じなかったことが示唆された。今回の多職種連携教育は臨床現場で行ったものではないことから、学生は専門性を感じにくかったのかもしれない。専門性が高まったと感じられるような授業に変更する必要があると考える。

授業前後の RIPLS の得点変化を対面と遠隔授業で比較したところ、全体合計得点の上昇は遠隔授業に比べて対面授業の方が有意に高かった。また、教員からの回答で「対面授業の方がよい」と答えた者の割合が多かったことから、総合的にみると対面授業の方が多職種連携教育の効果は高い可能性があると考える。授業前後の全体合計の得点変化は、「チームワークとコラボレーション」では、2021・2022 年度の遠隔授業では対面授業よりも有意に小さかったが、2023 年度の対面授業と遠隔授業の間では有意差を認めなかった。我々が 2021～2023 年度に行った遠隔授業は、教員間の議論に基づいて毎年改訂を行ってきたため、全く同じ内容ではない。ブラッシュアップをした結果、2023 年度の遠隔授業では過去の対面授業と同様にチームワークとコラボレーションの向上を感じられるようになったのかもしれない。また、学生はコロナ禍を通じて他の科目の遠隔授業や討議の経験を蓄積しており、これらも影響した可能性が考えられる。今回の研究では、先の結果で述べた遠隔授業のメリットも明らかになった。教員の関わりを強化し、学生達がチーム内で互いに学び合うことを促進する内容へとさらにブラッシュアップすることにより「チームワークとコラボレーション」においては対面授業と同等の教育効果が見込めるかもしれない。CAIPE (The Centre for the Advancement of Interprofessional Education)[15]によると、IPE は,「複数の領域の専門職種が連携およびケアの質を改善するために，二種類以上の異なる専門職種が同じ場所でともに学び、お互いから学び合いながら、お互いのことを学ぶこと」と定義しており、チームワークにはチームメンバー間の相互作用的な交流がかかせない[16]。「専門性」が遠隔授業よりも対面授業の方が得点上昇が有意に高いと検出されたところもあったが、実際の得点上昇値が極わずかであったことから、対面授業の方が専門性が高まったとは結論づけられない。

本研究では教育効果を RIPLS 日本語版で評価した。尺度の開発者により、Cronbach’s α係数は 0.74 で一定の信頼性を示していること、および因子分析の結果、「チームワークとコラボレーション」13 項目、「IPE の機会」2 項目、「専門性」4 項目の 3 つの下位尺度から構成されることが報告された（Tamura,2012）。しかし、その後の研究において、この尺度の信頼性・妥当性については、開発者が提唱する通りではないとの指摘が報告された。Aizawa ら[17]は多職種連携教育を受けた 3 学部学生を対象とした研究で、RIPLS 日本語版は下位尺度「専門性」の Cronbach’s α係数が低いことや、「IPE の機会」「専門性」が互いに独立した構造にならないといった構成概念妥当性の問題を指摘した。他言語版の RIPLS においても一部の下位尺度の信頼性が低いこと、開発者の提唱する通りの構成概念妥当性が得られないことがあること、天井効果があることが指摘されている[18-20]。多職種連携教育の効果を評価する指標としては、多職種連携能力を図るために開発された Chiba Interprofessional Competency Scale (CICS29)[21]など他の指標が有効である可能性があるため、今後の研究においてはそれらの使用を検討する必要があると考える。

対面・遠隔授業のメリット・デメリット

対面授業のメリットとして、【コミュニケーション・議論が円滑】【集中力・参加意識の向上】【チームの一体感】【他のチームからの刺激】【教員による観察・情報交換・支援が容易】のカテゴリーがあがったことから、対面授業ではチーム内で高い参加意識を持って集中してコミュニケーション・議論が行われていたことが窺われる。また、他のチームからの刺激や教員から支援を

得ながら進めていたと思われる。それに対して遠隔授業では【コミュニケーション・議論が円滑でない】【他のチームの状況を把握しづらい】【教員が介入しづらい】といったコミュニケーション上の問題が含まれているために、遠隔授業よりも対面授業の方が効果は高いと答えた教員が多かったと思われる。先行研究において、遠隔授業ではコミュニケーションが不十分となりやすいことが報告されている[22]。多職種連携教育において、参加者相互の議論や交流が重要であるため、遠隔授業で多職種連携教育を実施する場合にはコミュニケーション上の問題があることを踏まえる必要がある。一方、遠隔授業では【移動・コスト・場所の確保が不要なために参加しやすい】【準備・運営の負担が小さい】【チーム内討論に集中】【教員がチーム内の議論の状況を把握でき、評価が適切】【感染リスクがない】というメリットがあることが明らかになったことから、距離が離れた大学間を繋いで教員が関わりながらチーム内討論し、評価・改善を繰り返すことにより教育効果が発揮される可能性があると考える。今後、対面・遠隔授業それぞれのメリット・デメリットを理解し、メリットを活かした多職種連携教育の授業構築をすることが重要であると考える。

研究の限界

本研究におけるアンケート調査は自己評価であるため、客観性に問題があることは否めない。しかしながら、授業前後は同じ学生による評価を行っているので、前後比較には影響がないと考える。

対面授業は 2 日間、遠隔授業は 3 日間での実施であった。遠隔授業ではネットワークリテラシーの説明を行う必要があり遠隔授業の方が期間は長くなった。どの年度も「QOL」のテーマおよび TBL の手法で行われたため、比較する上での影響は少ないと考える。

今回の調査において、遠隔授業における学生からの回収率が 65.2%～80.1%と低かったことから選択バイアスが生じている可能性がある。教員からの回収率はほぼ半数に留まっており、対面・遠隔授業のメリット・デメリットが十分収集できていないかもしれない。

本研究ではアセンブリⅢの授業前後の比較を行っており、他の授業等の経験が結果に影響した可能性は否定できない。

結論

・全ての年度において授業前に比べて RIPLS 全体合計得点が有意に上昇したこと、下位尺度「チームワークとコラボレーション」の得点が有意に上昇したことから、対面・遠隔授業共に参加学生のチームワークとコラボレーションを高める効果があったことが示唆された。

・「IPE の機会」は対面授業で有意な上昇を認めたことから、我々が行った大規模な対面による多職種連携では、学生は IPE の機会が増えたと感じた可能性がある。

・「専門性」は対面でも遠隔でも授業前後でほとんど変化しなかったことから、対面・遠隔授業共に専門性を高めるための対策が必要である。

・遠隔授業に比べて対面授業の年度の方が授業前後の RIPLS 全体合計得点の上昇が有意に高かったこと、教員からの回答において「対面授業の方がよい」と答えた者の割合が多かったことから、総合的にみると対面授業の方が効果は高い可能性があることが示唆された。

・「チームワークとコラボレーション」の得点変化は 2021・2022 年の遠隔授業では対面授業よりも有意に小さかったが、2023 年の遠隔授業は対面授業との間で有意差を認めなかった。このことから、遠隔授業をさらにブラッシュアップすることにより「チームワークとコラボレーション」においては対面授業と同等の教育効果が見込めるかもしれない。
・教員が考える対面授業のメリットは【コミュニケーション・議論が円滑】【集中力・参加意識の向上】【チームの一体感】【他のチームからの刺激】【教員による観察・情報交換・支援が容易】であった。デメリットは、【移動に時間・コストがかかる】【大人数収容可能な教室の確保が困難】【準備・運営の負担が大きい】【他のチームの音で討論内容が聞こえづらい】【参加意識の低い人が視界に入ると悪影響】【教員が討論内容を把握しづらく評価が困難】であった。
・遠隔授業では【移動・コスト・場所の確保が不要なために参加しやすい】【準備・運営の負担が小さい】【チーム内討論に集中】【教員がチームの議論の状況を把握でき、評価が適切】【感染リスクがない】というメリットがある一方で、【コミュニケーション・議論が円滑でない】【集中しにくい】【他のチームの状況を把握しづらい】【教員が介入しづらい】【通信トラブル時の対応が困難】といったデメリットがあげられた。

謝辞
　本研究にご協力くださった全ての参加者に感謝申し上げます。

利益相反
　演題発表に関連し、開示すべき COI 関係にある企業・組織および団体等はありません。

References
1.Bluteau P, Jackson A. Interprofessional education. Basingstoke: Palgrave Macmillan; 2009: 37-58.
2.Angel VM, Friedman MH, Friedman AL. Integrating bar-code medication administration competencies in the curriculum: implications for nursing education and interprofessional collaboration. Nurs Educ Perspect 2016; 37: 239-41.
3.Nakamura S, Ohtsuki M, Miki Y, Noda T, Suzuki S, Maeno T, Matsui T. Effect of team-based learning in interprofessional education at a health university. Fujita Med J 2017;3:33-9.
4.Miyamoto M, Ohtsuki M, Rumi Seko, Nakamura S, Yano H, Suzuki S, Matsui T. Effects of community-oriented education using team-based learning on students' motivation to practice community health care. Fujita Med J 2017;3:28-32.
5.Nakamura S, Itoh M, Miki Y, Kido T, Kamei H, Suzuki S, Ohtsuki M. Relationship between peer evaluation and interprofessional self-evaluation in a joint healthcare team-based learning class involving three universities. Fujita Med J 2020 6,102-9.
6.Saguchi K, Okubo S, Kato R, Mitsuhashi K, Tanaka S, Kenmotsu S, Yoshikawa A, Enokida M. Students' evaluation of face-to-face and deostance teaching practices in a Multidisciplinary education program. Jaipe 2024; 17:134-45 (in Japanese).

7.Saitoh A, Yokono T, Sumiyoshi T, Kawachi I, Uchiyama M. A comparative study of face-to-face and online interprofessional education models for nursing students in Japan: A cross-sectional survey. Education Sciences 2023;13:1-11.
8.Michaelsen LK, Knight AB, Fink LD. Team-based learning: a transformative use of small groups in college teaching. Sterling, VA: Stylus Publishing; 2004: 3-207.
9.Das S, Nandi K, Baruah P, Sarkar SK, Goswami B, Koner BC. Is learning outcome after team based learning influenced by gender and academic standing? Biochem Mol Educ 2019; 47: 58–66.
10.Wahawisan J, Salazar M, Walters R, Alkhateeb FM, Attarabeen O. Reliability assessment of a peer evaluation instrument in a team based learning course. Pharm Pract (Granada) 2016; 14: 676.
11.Tamura Y, Seki K, Usami M, Taku S, Bontje P, Ando H, Taru C, Ishikawa Y. Cultural adaptation and validating a Japanese version of the readiness for interprofessional learning scale (RIPLS). J Interprof Care 2012;26:56-63.
12.Nagata Y, Yoshida M.Toukeiteki taju hikakuhou no kiso (Basics of Statistical Multiple Comparison Methods). Tokyo: Scientist; 2015:81-7 (in Japanese).
13.Shintani A. Kyou kara tsukaeru iryou toukei (Medical statistics you can use today). Tokyo: Igakushoin: 2015: 79-91 (in Japanese).
14.McMahon KK. Team formation. In: Team-based Learning for Health Professions education. Virginia: Stylus Pub; 2008:85-8.
15.The Centre for the Advancement of Interprofessional Education. CAIPE's definition of interprofessional education;2002
<https://www.caipe.org/> (Accessed Sep. 8. 2025).
16. Saras E., Sims DE., Burke CS. Is there a "BIG FIVE" in teamwork?. Small Group Research 2005;36: 555-99.
17.Aizawa F, Fujisawa M, Sato Y. A consideration of the application of the Japanese version of the readiness for interprofessional learning scale（RIPLS）：Analyzing questionnaire survey results from" Team Medical Literacy". Annual Report of Iwate Medical University Center for Liberal Arts and Sciences 2018; 53: 29-38 (in Japanese).
18.Mahler C, Berger S, Reeves S. The Readiness for Interprofessional Learning Scale (RIPLS): A problematic evaluative scale for the interprofessional field. J Interprof Care 2015;29:289-91.
19.Kerry MJ, Wang R, Bai J. Assessment of the Readiness for Interprofessional Learning Scale (RIPLS): An item response theory analysis. J Interprof Care 2018;32:634-7.
20.Torsvik M, Johnsen HC, Lillebo B, Reinaas LO, Vaag JR. Has "The Ceiling" Rendered the Readiness for Interprofessional Learning Scale (RIPLS) Outdated? J Multidiscip Healthc 2021;14:523-31.
21.Sakai I, Yamamoto T, Takahashi Y, Maeda T, Kunii Y, Kurokochi K. Development of a new measurement scale for interprofessional collaborative competency: The Chiba Interprofessional Competency Scale (CICS29). J Interprof Care 2017;31:59-65.
22.Sato N, Asada Y, Syuto T, Karino K. Shimyureshon supesharisuto kai ni okeru COVID-19 zengo no

katudou hikaku to ICT katsuyou no tenbou (Comparison of Pre- and Post-COVID-19 Activities and Prospects for ICT Utilization in the Simulation Specialist Group). Medical Education 2023; 54:657-9 (in Japanese).

Table1 Main learning structure and contents for each year in interprofessional education

Face-to-face learning in 2019	Day1	iRAT・tRAT（type of occupation）, appeals, feedback, application activities"How do patients wish to live?" and "Thinkning from the perspective of the patients and their family about what can be done to help them realize the way they wish to live.", Presentations and discussions
	Day2	application activities “Thinking about support for patients who have relapsed” and “Thinking about the role of the professional you aspire to be”, presentation and discussion, peer evaluation
	Day3	none
Distance learning in 2021	Day1	Review of Assembly I and II, network literacy
	Day2	iRAT・tRAT（type of occupation）, appeals, feedback, application activities"How do patients wish to live?" and "Thinkning from the perspective of the patients and their family about what can be done to help them realize the way they wish to live.", Presentations and discussions
	Day3	iRAT・tRAT（QOL, ACP: Advance care planning）, appeals, feedback, application activities"Imagining the feels of a patient who has relapsed and thinking about what you can do to alleviate their anxiety.", peer evaluation
Distance learning in 2022	Day1	network literacy, iRAT・tRAT（type of occupation, QOL）、appeals, feedback, application activities"Thinking about how you would feel if you were a patient or family member", presentation and discussion
	Day2	iRAT・tRAT（ACP, COVID-19）, appeals, feedback, application activities"Thinking from the perspective of those around the patient", " Considering the feelings of the patient who hase been undergoing treatment."
	Day3	presentation and discussion, peer evaluation
Distance learning in 2023	Day1	network literacy, iRAT・tRAT（type of occupation）appeals, feedback, application activities, "Thinking about how you would feel if you were a patient or family member", and "Thinking from the perspective of those around the patient", presentation and discussion
	Day2	iRAT・tRAT（QOL, ACP）, appeals, feedback, application activities" Considering the feelings of the patient who hase been undergoing treatment.", and "Thinking about what each profession can do for patients, their families, and those around them"
	Day3	presentation and discussion, peer evaluation

Table2 Attributes of the subjects (students)

year	2019			2021			2022			2023		
Department	Faculty and grade	n	%	Faculty and grade	n	%	Faculty and grade	n	%	Faculty and grade	n	%
Medicine	Medicine, grade 3	107	12.8	Medicine, grade 3	75	9.7	Medicine, grade 3	100	13.1	Medicine, grade 3	90	13.5
Medical sciences	Medical technology, grade3	96	11.5	Medical technology, grade3 (including medical technology program and clinical engineering program)	102	13.1	Medical technology, grade3 (including medical technology program and clinical engineering program)	101	13.1	Medical technology, grade3 (including medical technology program and clinical engineering program)	90	13.5
	Clinical engineering, garade3	47	5.6									
	Radiological technology, grade3	59	7.0	Radiological technology, grade3	71	9.1	Radiological technology, grade3	66	8.6	Radiological technology, grade3	60	9.0
	Medical Management and information science, grade4	32	3.8	Medical Management and information science, grade4	33	4.2						
Health sciences	Nursing, grade3	136	16.2	Nursing, grade3	99	12.7	Nursing, grade3	108	14.1	Nursing, grade3	80	12.0
	Rehabilitation, grade3	52	6.2	Rehabilitation, grade3	90	11.6	Rehabilitation, grade3	83	10.9	Rehabilitation, grade3	76	11.5
Pharmacy	Pharmacy, grade4	244	29.2	Pharmacy, grade4	203	26.1	Pharmacy, grade4	207	27.0	Pharmacy, grade4	181	27.2
Social welfare	Social welfare, garade3, 4	34	4.1	Social welfare, garade3, 4	8	1.0	Social welfare, garade3, 4	26	3.4	Social welfare, garade3	8	1.2
Dentistry	Dentistry, grade6	22	2.6	Dentistry, grade3	91	11.7	Dentistry, grade3	66	8.6	Dentistry, grade3	74	11.1
Psychological and Physical Science	Health nutrition, grade4	8	1.0	Health nutrition, grade4	5	0.6	Health nutrition, grade4	9	1.2	Health nutrition, grade4	7	1.1
	Total	837	100	Total	777	100	Total	766	100	Total	666	100

Table3 Attributes of the subjects (teachers)

Department	n	%
Medicine	4	9.7
Medical sciences	9	22.0
Health sciences	11	26.8
Pharmacy	9	22.0
Social welfare	2	4.9
Dentistry	5	12.2
Psychological and Physical Science	1	2.4
Total	41	100.0

Table4 Comparison of RIPLS scores before and after class

		Face-to-face learning in 2019	Distance learning in 2021	Distance learning in 2022	Distance learning in 2023
		(n=837)	(n=777)	(n=766)	(n=666)
		Mean±SD	Mean±SD	Mean±SD	Mean±SD
Teamwork and collaboration (13 items)	Before class	50.4±5.9	52.4±5.8	51.8±6.2	52.1±6.5
	After class	52.9±6.2	53.6±6.6	53.1±6.4	54.1±6.6
	Difference in scores before and after class[1]	2.5	1.2	1.3	2.0
	p value	<0.001	<0.001	<0.001	<0.001
IPE opportunities (2 items)	Before class	6.9±2.0	7.8±1.9	7.6±2.0	7.5±2.0
	After class	7.3±2.2	7.7±2.1	7.5±2.2	7.5±2.3
	Difference in scores before and after class[1]	0.4	-0.1	-0.1	0.0
	p value	<0.001	0.861	0.484	0.725
Uniqueness of profession (4 items)	Before class	12.4±2.0	13.0±1.8	12.8±2.0	12.9±2.0
	After class	12.6±2.1	12.9±2.1	12.8±2.1	12.9±2.3
	Difference in scores before and after class[1]	0.2	-0.1	0.0	0.0
	p value	0.003	0.444	0.524	0.956
Overall (19items)	Before class	69.7±7.5	73.1±7.5	72.1±7.8	72.5±8.3
	After class	72.8±7.9	74.2±8.5	73.4±8.1	74.5±8.6
	Difference in scores before and after class[1]	3.1	1.1	1.3	2.0
	p value	<0.001	<0.001	<0.001	<0.001

Wilcoxon signed rank test (Significant difference at $p < 0.05/16 = 0.003125$ for multiplicity with Bonferroni correction)

SD, standard deviation

[1]"after class scores" minus "before class scores"

Table5 Comparison of scores before and after class differences between 2019 face-to-face classes and other years' distance learning

	Face-to-face learning in 2019 (n=837)	Distance learning in 2021 (n=777)	Comparison between 2019 and 2021	Distance learning in 2022 (n=766)	Comparison between 2019 and 2022	Distance learning in 2023 (n=666)	Comparison between 2019 and 2023
	Difference in scores before and after class[1]	Difference in scores before and after class[1]	p value	Difference in scores before and after class[1]	p value	Difference in scores before and after class[1]	p value
Teamwork and collaboration (13 items)	2.5	1.2	<0.001	1.3	<0.001	2.0	0.095
IPE opportunities (2 items)	0.4	-0.1	<0.001	-0.1	<0.001	0.0	<0.001
Uniqueness of profession (4 items)	0.2	-0.1	0.004	0.0	0.104	0.0	0.068
Overall (19items)	3.1	1.1	<0.001	1.3	<0.001	2.0	0.001

Mann-whitney test (Significant difference at $p<0.05/12 = 0.004166$ for multiplicity with Bonferroni correction)

[1]"after class scores" minus "before class scores"

Table6 Whether face-to-face or distance learning is preferable

	n	%
Face-to-face learning	29	70.7
Distance learning	2	4.9
Neither	10	24.4
Total	41	100

Table7 Advantages and disadvantages of face-to-face and distance learning (categories)

	Advantages	Disadvantages
Face-to-face learning	Smooth communication and discussion<br>Improved concentration and sense of participation<br>Sense of team unity<br>Stimulation from other teams<br>Easy observation, exchange of information, and support by teachers	Time-consuming and costly to move<br>Difficult to secure a classroom that can accommodate large numbers of people<br>Heavy burden of preparation and administration<br>Difficult to hear discussion content through other teams' voices<br>Negative impact when people with low awareness of participation are in view<br>Difficult for teachers to grasp and evaluate discussion content
Distance learning	Easier to participate due to no need for movement, cost, or location<br>Less burden of preparation and administration<br>Focus on intra-team discussions<br>Teachers can monitor team discussions and evaluate appropriately<br>No risk of infection	Communication and discussion are not smooth<br>Difficult to stay focused<br>Difficult to grasp the situation of other teams<br>Difficult for teachers to intervene<br>Difficult to respond to communication failures
